# Depression, sleep disturbances, pain, disability and quality of LIFE in Brazilian Fabry disease patients

**DOI:** 10.1016/j.ymgmr.2019.100547

**Published:** 2019-12-02

**Authors:** Nilton Salles Rosa Neto, Judith Campos de Barros Bento, Rosa Maria Rodrigues Pereira

**Affiliations:** Rheumatology Division, Faculdade de Medicina da Universidade de São Paulo, São Paulo, Brazil

**Keywords:** Fabry disease, Patient-reported outcomes, Depression, Sleep disturbances, Disability, Quality of life, FD, Fabry disease, ERT, enzyme replacement therapy, M, male, F, female, MSSI, Mainz Symptom Severity Index, QoL, Quality of Life, PROs, patient-reported outcomes, HR-QoL, health-related quality of life, HAM-D, Hamilton Depression Rating Scale, PSQI, Pittsburgh Sleep Quality Index, BPI, Brief Pain Inventory, HAQ-DI, Health Assessment Questionnaire Disability Index, SF-36, Short-Form Health Survey 36, QALYs, quality adjusted life years, SD, standard deviation

## Abstract

Background: Fabry disease (FD) is a lysosomal disease in which mutations affect the *GLA* gene located on the X chromosome. The defective product, the enzyme alpha-galactosidase A, causes accumulation of substrate and contributes to the disruption of cell function in several organs, with variable severity and consequent damage of tissue or organ function. Patient reported outcomes (PROs) enable patients to provide information regarding the consequences of their disease and its treatment and are often recognized as the most important outcomes for them.

Objectives: To evaluate pain, depression, sleep disturbances, disability and disease impact on quality of life in a cohort of Brazilian FD patients and compare between groups stratified by the Mainz Symptom Severity Index (MSSI) Methods: Thirty-seven genotype confirmed classic FD patients – 16 male and 21 female – (mutations: C142R, A156D, L180F, R227X, W262X, G271A, P293S, Y264SX) were evaluated and answered the following questionnaires: Brief Pain Inventory (BPI), Hamilton Depression Rating Scale (HAM-D), Pittsburgh Sleep Quality Index (PSQI), Health Assessment Questionnaire Disability Index (HAQ-DI), Short-Form Health Survey 36 (SF-36).

Results: In FD patients, mean ± SD BPI severity result was 2.78 ± 2.66 for severe; 2.80 ± 2.55 for moderate and 1.55 ± 2.38 for mild severity patients. Mean ± SD BPI interference result was 2.55 ± 2.44 for severe; 2.80 ± 3.18 for moderate and 1.36 ± 2.83 for mild patients. BPI severity and interference values correlated with MSSI scores (*r* = 0.24; *p* < .001 / *r* = 0.25; p < .001). Application of HAM-D indicated depression in 21 patients (56.8%). HAM-D results had positive correlation with MSSI values (*r* = 0.21; *p* < .001), with BPI severity (*r* = 0.54; *p* < .001) and interference (*r* = 0.65; p < .001). PSQI depicted sleep disturbances in 22 patients (59.5%). PSQI values correlated with MSSI values (*r* = 0.25; *p* < .001), with HAM-D results (*r* = 0.65; *p* < .001) and BPI severity (*r* = 0.47; *p* < .001) and interference (*r* = 0.66; *p* < .001). Mean HAQ-DI result was 0.490 for severe; 0.274 for moderate and 0.157 for mild severity patients. Conclusions: Depression, sleep disturbances and disability were under-recognized in FD patients. HAQ-DI revealed worse disability according to MSSI severity status. The lowest raw scores from the SF-36 questionnaire were for the domains general health perception and physical role functioning. Standardized assessments should be routine care and started as early as diagnosis of Fabry disease is made.

## 1 Background

Fabry disease (FD) is a lysosomal disease in which mutations affect the *GLA* gene located on the X chromosome. The defective product, the enzyme alpha-galactosidase A, causes accumulation of substrate that contributes to the disruption of cell function in several organs, with variable severity and consequent damage of tissue or organ function [1,2].

Being a progressive storage disease with patients presenting chronic kidney disease, cardiomyopathy, stroke, and perhaps requirement of transplantation, FD patients are known to have more depression, chronic pain, worse sleep quality, functionality and quality of life. In this sense, health-related quality of life (HR-QoL) is reduced in FD patients due to both somatic and psychological impairment [3,4]. Of note, psychiatric symptoms, particularly depression, are highly prevalent in men and women with FD [3,5,6].

Factors associated with lower QoL include Fabry-related chronic pain and pain crises, gastrointestinal symptoms, hearing loss, physical inactivity and fatigue. Important, specific organ damage, such as kidney failure, also severely affects QoL [4,7]. Poorer QoL in patients with FD is also related to phenotype, age and disease severity. Classically affected patients of older age will have more severe disease with a higher chance of developing FD-related complications and, therefore, resulting in decreased QoL [8].

Patient-reported outcomes (PROs) state the patient's health condition directly from the patient. PROs enable patients to provide information regarding the consequences of their disease and its treatment and are often recognized as the most important outcomes for them. In FD studies, Short-Form Health Survey 36 (SF-36) is the most applied questionnaire and pain is the symptom most commonly assessed [9].

## 2 Objectives

To evaluate pain, depression, sleep disturbances, disability and disease impact on quality of life in a cohort of Brazilian FD patients and compare between groups stratified by the Mainz Symptom Severity Index (MSSI) [10].

## 3 Methods

Thirty-seven genotype confirmed classic FD patients – 16 male and 21 female – (mutations: C142R, A156D, L180F, R227X, W262X, G271A, P293S, Y264SX) were interviewed by the same researcher (NSRN) and had clinical, laboratory and imaging data reviewed followed by the application of the questionnaires.

Brief Pain Inventory (BPI) consists of a 17-item self-report questionnaire addressing intensity, quality, relief and interference of pain [11,12]. Patients are asked to score items using a numeric scale from 0 to 10. Hamilton Depression Rating Scale (HAM-D) 21 items scale is scored between 0 and 4 points. The score is based on the 17 first items that measure severity of depressive symptoms. Scores from 0 to 7 are considered normal, 8 to 16: mild depression, 17 to 23: moderate depression, and scores above 24: severe depression [13,14]. Pittsburgh Sleep Quality Index (PSQI) is an instrument that measures quality and sleep patterns by analyzing seven domains: subjective sleep quality, sleep latency, sleep duration, habitual sleep efficiency, sleep disturbance, use of sleeping medication and daytime dysfunction in the last month [15,16]. Patients score based on a scale from 0 to 3, where 3 is the negative extreme**.** Scoring discriminates sleep dysfunction as good sleepers (PSQI 0–5 points) or poor sleepers (PSQI >5 points)**.** Health Assessment Questionnaire Disability Index (HAQ-DI) is a self-administered questionnaire originally developed for rheumatoid arthritis that assesses categories such as dressing and grooming; arising; eating; walking; hygiene; reach; grip; and common daily activities [17,18]. The choice of selecting HAQ-DI, a questionnaire used commonly in rheumatology, for assessment of functionality in FD patients, was due to the fact that many patients complained of arthralgia or arthritis and also to assess feasibility of comparison of disability in FD and rheumatic diseases. HAQ-DI index can be interpreted as 0–1: mild difficulties to moderate disability; 1–2: disability moderate to severe, and 2–3: severe to very severe disability. Short-Form Health Survey 36 (SF-36) consists of eight scaled scores that measure quality of life (vitality; physical functioning; bodily pain; general health perceptions; physical role functioning; emotional role functioning; social role functioning; and mental health). Each scale is directly transformed into a scale from 0 to 100 where the lower the score means greater disability [19,20]. All questionnaires were adapted and validated to Brazilian Portuguese.

### 3.1 Statistical analysis

Results are presented as mean and standard deviation for continuous variables and percentages for categorical variables. Correlation between continuous variables was measured by Pearson's correlation coefficient. We considered significant a *p*-value < .05.

## 4 Results

Mean ± SD age of patients overall was 43.1 ± 15.4 years (male 44.4 ± 11.6 years and female 42.1 ± 17.7). Twenty-two patients (59.5%) received enzyme replacement therapies – agalsidase alfa (*n* = 17) and agalsidase beta (*n* = 5).

### 4.1 Pain

FD patients were stratified according to MSSI status as severe, moderate or mild.

In FD patients, mean ± SD BPI severity result was 2.78 ± 2.66 for severe; 2.80 ± 2.55 for moderate and 1.55 ± 2.38 for mild severity patients. Mean ± SD BPI interference result was 2.55 ± 2.44 for severe; 2.80 ± 3.18 for moderate and 1.36 ± 2.83 for mild patients. BPI severity values correlated with MSSI scores (*r* = 0.24; *p* < .001) as well as BPI interference (*r* = 0.25; p < .001).

Over-the-counter pain medication was used by 22 subjects (59.5%). Opioid was use by 3 patients: codein+acetaminophen (*n* = 1), codein or tramadol (*n* = 1) and oxycodone (*n* = 1). Anticonvulsants and/or antipsychotic drugs were used as pain modulators by 6 patients (16.2%): carbamazepine (*n* = 1); carbamazepine + gabapentin (*n* = 1); gabapentin (*n* = 1); pregabalin (*n* = 1); pregabalin + chlorpromazine (*n* = 1); chlorpromazine (*n* = 1).

### 4.2 Depression

After medical interview, depression was referred by 11 patients (29.7%). Application of HAM-D indicated depression in 21 patients (56.8%). Twelve patients were classified as mild; 4 patients as moderate, and 5 patients as having severe depression. HAM-D results had positive correlation with MSSI values (*r* = 0.21; *p* < .001), with BPI severity (*r* = 0.54; *p* < .001) and interference (*r* = 0.65; *p* < .001). Of note, only 6 patients (16.2%) were on antidepressants, one of them using two different drugs because of pain management: fluoxetine (*n* = 1); duloxetine (n = 1); sertraline (*n* = 2); venlafaxine (*n* = 1) and fluoxetine + amitriptyline (*n* = 1).

### 4.3 Sleep disturbances

Medical interview aimed at evaluating insomnia and/or unrefreshing sleep, which were reported by 17 FD patients (45.9%). PSQI depicted sleep disturbances in 22 patients (59.5%). PSQI values correlated with MSSI values (*r* = 0.25; *p* < .001), with HAM-D results (*r* = 0.65; p < .001) and BPI severity (*r* = 0.47; p < .001) and interference (*r* = 0.66; *p* < .001). Three patients were in therapy with hypnotics: alprazolam (*n* = 2) and clonazepam (*n* = 1).

### 4.4 Disability

FD patients were stratified according to MSSI status as severe, moderate or mild.

Mean HAQ-DI result was 0.490 for severe; 0.274 for moderate and 0.157 for mild severity patients. HAQ-DI values ranged from 0 to 1.250. Only one patient had a HAQ-DI score above 1 (a female A156D patient considered to have mild disease scored 1.250 – there was chronic pain but no kidney, heart or central nervous system damage neither there was arthritis). HAQ-DI values correlated with MSSI scores (*r* = 0.45; *p* < .001); with HAM-D values (*r* = 0.34; *p* < .001), with PSQI values (*r* = 0.43; p < .001), with BPI severity (*r* = 0.54; p < .001) and BPI interference (*r* = 0.59; p < .001).

### 4.5 Quality of life

SF-36 raw scores (lower the score, greater the disability) of FD patients are shown in Table 1. The lowest scores (mean ± SD) overall in this cohort were general health perception (48.89 ± 21.03) and physical role functioning (51.35 ± 39.40). When comparing groups by sex, general health perception is poorer for male patients.

**Table 1:** SF-36 raw score results.

SF-36 concepts	FD patients (*N* = 37)	Female patients (*N* = 21)	Male patients (*N* = 16)	p-value
	Mean ± SD	Mean ± SD	Mean ± SD	
Vitality	54.05 ± 20.53	58.8 ± 19.0	47.8 ± 20.8	0.11
Physical functioning	56.08 ± 24.75	61.9 ± 25.6	48.4 ± 21.3	0.11
Bodily pain	62.30 ± 23.47	59.2 ± 22.3	66.4 ± 24.3	0.37
General health perceptions	48.89 ± 21.03	56.4 ± 20.7	39.1 ± 17.1	**0.01**
Physical role functioning	51.35 ± 39.40	52.4 ± 40.0	50.0 ± 38.5	0.86
Emotional role functioning	53.15 ± 42.06	50.8 ± 40.7	56.3 ± 43.7	0.71
Social role functioning	71.28 ± 25.48	73.2 ± 26.5	68.8 ± 23.8	0.61
Mental health	66.59 ± 22.48	68.8 ± 23.5	63.6 ± 20.8	0.52

SD: Standard deviation. Difference of SF-36 raw score between female and male patients is statistically significant.

In FD patients, SF-36 scores in physical functioning and physical role functioning correlated with HAQ-DI scores; bodily pain scores correlated with both BPI Severity and Interference scores; mental health component correlated with HAM-D score; and general health perceptions correlated with MSSI score as seen in Table 2**.**

**Table 2:** Correlation of SF-36 results.

Concepts	FD patients	
	Pearson's coefficient	p-value
Physical functioning × HAQ-DI	−0.63	p < .001
Physical role functioning × HAQ-DI	−0.53	p < .001
Bodily pain × BPI severity	−0.78	p < .001
Bodily pain × BPI interference	−0.70	p < .001
General health perceptions × MSSI score	−0.29	p < .001
Mental health × HAM-D	−0.77	p < .001

HAQ-DI: Health Assessment Questionnaire – Disability index; BPI: Brief Pain Inventory; MSSI: Mainz Symptom Severity Index; HAM-D: Hamilton Depression Rating Scale.

## 5 Discussion

Patient-reported outcomes observing physical and mental health issues as well as functionality and quality of life are considered central aspects of health surveillance and indicators of requirements and outcomes of treatment [21]. Several countries are demanding appraisal of quality adjusted life years (QALYs) to obtain costs estimation and cost-effectiveness analysis in order to support reimbursement decisions [4].

Health-related quality of life (HR-QoL) is a multidimensional concept referring to patient's insights of the impact of both disease and treatment on physical, psychological and social functions and well-being without the observer's bias [21]. The use of structured instruments during follow-up of treated and non-treated patients improves this knowledge and optimizes diagnosis and, hence, patients´ QoL.

This study emphasizes the presence of pain as assessed by BPI in FD patients; the elevated use of over-the-counter analgesics and the low prescription of pain modulators. Pain scores correlated with depression, sleep disturbances and quality of life scores and should be routinely measured in order to reduce its impact on other aspects of the disease.

Depression is the most frequently reported psychiatric complication of FD ranging from 15 to 62% prevalence and can significantly affect disease burden [5,6]. Furthermore, depression is under-recognized in the general population [22]. Lohle et al. [23] found that almost half of FD subjects reporting severe depression were undiagnosed and untreated depression. Our results reflect the same findings, with under-recognition of depression, and also under-treatment of FD patients. This may be related to the fact that most patients in this cohort are not followed in multidisciplinary centers or do not have access to specialized care with knowledge about the disease.

Patients whose depression is associated with chronic conditions can cope more effectively with their illness and symptoms if their depression is treated [22]. Depression is related to elevated risk of suicide, and higher mortality, impaired psychosocial function and reduced QoL [24]. Early and energetic treatment for depression improves outcome, decreases morbidity and mortality, enhances social and economic well-being for the individual and the family [22]. In this way, people caring for FD patients should maintain a high degree of clinical suspicion for depression in order to improve diagnosis and treatment [22]. In FD patients, depression correlated with MSSI scores reflecting the impact of disease burden.

Demographic studies suggested that sleep disorders, particularly sleep disordered breathing are common in FD patients. Obstructive sleep apnea exerts a detrimental effect on quality of life and excessive daytime sleepiness is higher than in controls. Previous studies reported a prevalence of 17 to 25% sleep disorders in FD patients [25]. Up to 68% of FD male patients have excessive daytime sleepiness [26]. Our results show that almost half of FD patients referred insomnia and/or unrefreshing sleep. Moreover, PSQI instrument improved detection of bad sleepers and was correlated with MSSI score. HAQ-DI was never assessed in FD patients before. Elevated HAQ-DI values meaning disability correlated with MSSI score in patients. FD patients considered to have severe disease had a mean HAQ-DI of 0.490. A number to aid in the interpretation of this result may be obtained from the REAL study where Brazilian rheumatoid arthritis patients were assessed in specialized centers throughout the country [27]. Mean HAQ-DI value in 1111 patients was 0.875 (ranging from 0 to 3), with mean disease duration 12.7 years; mean age 56.7 years (22.1–88.8) and marked female predominance (89.4%). Since FD patients may be seen by rheumatologists during their investigational odyssey when arthralgia is a chief symptom, it is interesting to point that their HAQ-DI values might overlap according to their severity. Careful history and physical examination alongside epidemiological data are required to aid in the differential diagnosis and proper treatment.

QoL in Brazilian FD patients was assessed in one study which included 14 FD patients and 21 Gaucher disease patients. General health scores were poorer in FD than in Gaucher disease patients [28]. In terms of SF-36 raw scores, our results were comparable to FD patients on ERT (*n* = 10) in their cohort. FD patients also scored worse on physical role functioning (45.0 ± 44.7) and general health perceptions (50.3 ± 26.8) [28].

Therapeutic goals for QoL comprise adequate management of pain and gastrointestinal symptoms and reduction of disease-associated morbidity due to target organ impairment. The diagnosis of FD followed by a treatment strategy may improve QoL in some patients only because of the psychological benefits of having a multidisciplinary team monitoring their health. It is important to take into consideration specific disease manifestations a patient might have, age and disease severity at the time of treatment initiation when assessing QoL scores [7]. To better detect changes related to interventions for rare lysosomal storage diseases, the development and use of disease-specific patient-reported outcomes are needed [9].

## 6 Conclusions

Depression, sleep disturbances and disability were under-recognized in FD patients in this cohort. HAQ-DI revealed worse disability according to MSSI severity status in patients. The lowest raw scores from the SF-36 questionnaire were general health perception and physical role functioning. General health status is poorer in male than female FD patients.

These results reinforce the need for psychiatric and psychosocial evaluations as standard care for Fabry disease patients as part of a multidisciplinary team. A basic assessment should include investigation of mood disorders, suicidality, suitability of pain control, functionality and quality of life. Assessments should be started as early as diagnosis of Fabry disease is made and should include children and adolescents. Timely intervention and care of specialized professionals may definitely improve outcome.

## Ethics approval and consent to participate

This work was approved by Faculdade de Medicina da Universidade de São Paulo Ethics Review Board under number 1.464.841 on March 24th 2016. All patients read and signed informed consent. The procedures followed were in accordance with the ethical standards of the responsible committee on human experimentation and with the Helsinki Declaration of 1975, as revised in 1983.

## Funding

FAPE – Brazilian Society of Rheumatology Research Fund Brazilian Society of Rheumatology had no role in the design of the study, collection, analysis and interpretation of data and in writing the manuscript.

## Declaration of Competing Interests

NSRN declares having received speaker´s and advisory board fees from Shire HGT, now Takeda Pharmaceuticals; JCBB declares that spouse received speaker´s and advisory board fees from Shire HGT, now Takeda Pharmaceuticals; RMRP has nothing to disclose.
